# Alveolar duct expansion greatly enhances aerosol deposition: a three-dimensional computational fluid dynamics study

**DOI:** 10.1098/rsta.2008.0295

**Published:** 2009-06-13

**Authors:** C. Darquenne, L. Harrington, G.K. Prisk

**Affiliations:** Department of Medicine, University of CaliforniaSan Diego, 9500 Gilman Drive, mail code 0931, La Jolla, CA 92093-0931, USA

**Keywords:** human acinus, computational modelling, aerosol transport, moving boundaries

## Abstract

Obtaining *in vivo* data of particle transport in the human lung is often difficult, if not impossible. Computational fluid dynamics (CFD) can provide detailed information on aerosol transport in realistic airway geometries. This paper provides a review of the key CFD studies of aerosol transport in the acinar region of the human lung. It also describes the first ever three-dimensional model of a single fully alveolated duct with moving boundaries allowing for the cyclic expansion and contraction that occurs during breathing. Studies of intra-acinar aerosol transport performed in models with stationary walls (SWs) showed that flow patterns were influenced by the geometric characteristics of the alveolar aperture, the presence of the alveolar septa contributed to the penetration of the particles into the lung periphery and there were large inhomogeneities in deposition patterns within the acinar structure. Recent studies have now used acinar models with moving walls. In these cases, particles penetrate the alveolar cavities not only as a result of sedimentation and diffusion but also as a result of convective transport, resulting in a much higher deposition prediction than that in SW models. Thus, models that fail to incorporate alveolar wall motions probably underestimate aerosol deposition in the acinar region of the lung.

## 1. Introduction

While it is widely accepted that fine particles (smaller than 2.5 μm) can penetrate deep into the alveolar region of the lung, no technique allowing for accurate direct *in vivo* measurements is yet available. Therefore, information on aerosol deposition patterns and mixing processes has relied on indirect experimental measurements and heavily on models of aerosol transport within the lung. Measurements *in vivo* are usually limited to the monitoring of inspired and expired particles at the mouth or to the use of radio-aerosols and imaging techniques to picture the gross patterns of aerosol deposition.

Computational fluid dynamics (CFD) has been increasingly used over recent years to study aerosol transport and deposition in the lung as the necessity of using multidimensional approaches for accurate predictions became apparent. Indeed, unlike the situation for gases, one-dimensional models are inaccurate for aerosol transport (Darquenne & Paiva 1996) because they rest on the implicit assumption that velocity and concentration are uniform over the cross section of the airways. Neither of these assumptions is valid for aerosols that have very low diffusivity and whose transport is mainly governed by convective effects, even in the very periphery of the lung. While there have been numerous CFD studies of aerosol transport in the conducting airways, few of these have focused on the alveolar region of the lung. The first of these latter studies used rigid-walled alveolar duct structure. Tsuda *et al*. (1994*a*,*b*) used an infinitely long axisymmetric alveolated duct. Darquenne & Paiva (1996) and Harrington *et al*. (2006) developed two- and three-dimensional models of alveolated ducts as well as a more complex two-dimensional multi-bifurcation structure of the acinar region of the lung (Darquenne 2001, 2002; Darquenne & Prisk 2003). More recently, Harrington *et al*. (2006) have developed the first three-dimensional model of a single bifurcation of alveolated ducts. All these studies showed that flow patterns were influenced by geometric characteristics, in particular at the level of the alveolar aperture, that the presence of the alveolar septa contributed to the penetration of the particles in the periphery of the lung, and that there were large inhomogeneities in deposition patterns within the acinar structure.

However, all of these studies were hampered by the fact that the simulations occurred in a rigid structure, while the lung itself rhythmically expands and contracts during breathing. As CFD techniques have developed, it is now feasible to actually consider models in which the walls of a complex structure move, and there have been a few studies in this area. Tsuda *et al*. (1995) showed that the flow pattern in a single expanding alveolus differed substantially from that in a rigid-walled model. The same group also developed an axisymmetric model of an alveolated duct with expanding walls comprising a central channel around which nine tori were placed (Henry *et al*. 2002). They showed that the peculiar geometry of the alveolated duct and its cyclic motion during breathing generated flow that exhibited multiple saddle points, characteristic of chaotic flow, resulting in substantial flow irreversibility. They suggested that this chaotic mixing might be the dominant mechanism of aerosol transport deep in the lung. Recently, Haber *et al*. (2003) have shown that deposition in a rhythmically expanding and contracting alveolus is much higher than that in a rigid-walled model.

The paper provides a detailed review of previous key CFD studies of aerosol transport and deposition in the acinar region of the lung. It also describes the first ever three-dimensional model of a single fully alveolated duct with moving boundaries allowing for the simulation of the cyclic expansion and contraction that occurs during breathing. Finally, predictions of aerosol deposition are compared with those obtained in a similar model with rigid walls.

## 2. Methods

### (a) Model geometry

**Figure 1:**
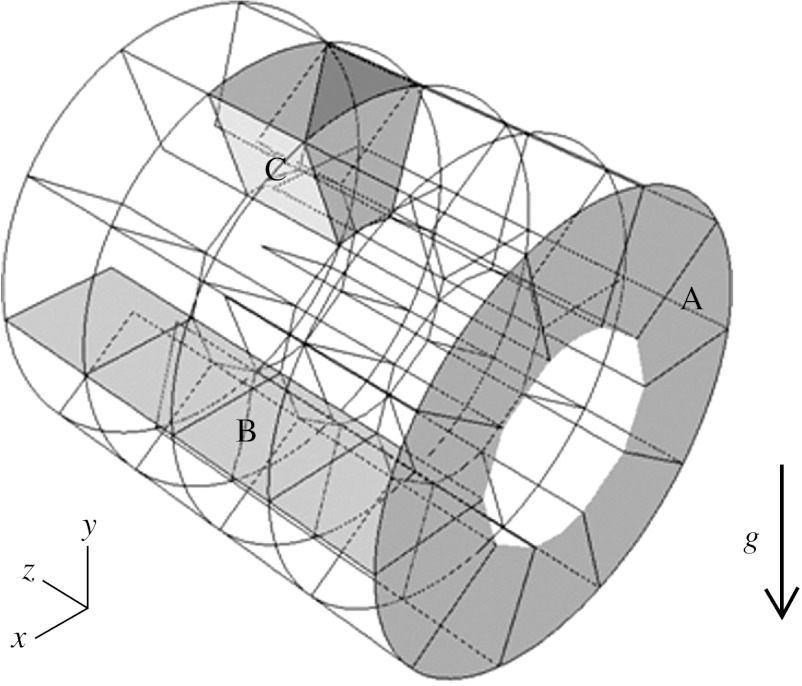
Duct model geometry. Axial planes (A) and radial planes (B) delineate the individual alveoli (C). Model dimensions at the beginning of inspiration are 267 μm for the lumen diameter, 606 μm for the outer diameter and 600 μm for the duct length. Length, average width and depth of the alveoli are 150×137×169.5 μm. Model is shown in horizontal position with gravity force (*g*) acting downwards.

The geometric characteristics of the model with moving walls (MWs) are similar to those of our previous three-dimensional model with rigid walls (Harrington *et al*. 2006). These characteristics are representative of realistic alveolar depth, alveolar width, duct length and lumen diameter based on morphometric data of Haefeli-Bleuer & Weibel (1988). While people often think of the lung as a structure in which each successive generation is a smaller copy of the parent (i.e. fractal), once the acinar region is reached, the structure size becomes much more constant as reflected in our model. Briefly, the model consisted of a cylindrical lumen surrounded by annuli divided into discrete alveoli (figure 1). The model dimensions were 267 μm for the lumen diameter, 606 μm for the outer diameter and 600 μm for the duct length. The model had four alveoli in the axial direction and 10 in the radial direction, giving an alveolar width of 137 μm and an alveolar length of 150 μm. As alveolar width varies over the height of the alveoli, the width dimension stated was measured at mid-alveolar depth.

### (b) Flow model

The flow field calculations were carried out by a general purpose CFD code (Star-cd v. 3.15, CD-Adapco) using the SIMPLE implicit finite volume solution algorithm with MARS second-order discretization (CD-Adapco 2001). The generated mesh used 135 000 hexahedral cells. Such a mesh size proved to provide accurate solutions for flow field and aerosol deposition (Harrington *et al*. 2006). The flow conditions in the alveolar region of the lung were fully represented by the laminar, isothermal and incompressible Navier–Stokes equations using the fluid properties of air at 37°C. A no-slip boundary condition was applied at the wall boundaries (i.e. the fluid has zero velocity relative to the wall). Owing to the laminar flow regime, the velocity profile at the inlet of the model was assumed to be parabolic. The gradients of all variables along the flow direction were set to be zero at the outlet of the model. An additional equation, the space conservation law, was solved for the moving coordinate velocity components (CD-Adapco 2001). This relates the change in cell volume to the coordinate frame velocity. Cell volume change was assumed to be isotropic. At every time step of the computation, each cell volume was multiplied by a constant factor corresponding to the volume increase between time steps.

Flow field was computed for inspiration in models corresponding to generations 18 (*Z*=18) and 23 (*Z*=23) of the Weibel symmetric lung model (Haefeli-Bleuer & Weibel 1988), representing the region near the entrance of the acinus and the most peripheral air sacs, respectively. Simulations were performed in the alveolated duct with both stationary wall (SW) boundaries and MW boundaries. In the MW structures (*Z*=18 and 23), the model was assumed to expand isotropically to 133 per cent of its original volume, typical of a tidal breath. The variation in the alveolar volume was shared equally between each alveolus. For *Z*=23, the distal end of the lumen was closed in the MW structure, and open in the SW structure to satisfy mass balance. A parabolic velocity profile was imposed at the inlet of the alveolated duct and was set to be representative of a flow rate at the mouth of 500 ml s^−1^ in all cases. For the MW alveolar sac (*Z*=23), flow was induced solely as a result of the motions of the alveolar walls. Flow was characterized by a maximum Reynolds number *Re*_max_ of 1.25 and 0.04 for *Z*=18 and 23, respectively, where *Re*_max_ was calculated based on the diameter of the lumen and the maximum velocity in the model.

### (c) Particle transport model

Particles ranging from 1 to 5 μm in diameter were considered in the study. Particles were introduced at the inlet of the model and were tracked while accounting for particle inertia, drag and gravitational sedimentation. Brownian diffusion was ignored as its effect is negligible for particles larger than 0.5 μm (Brain & Valberg 1979). The equation governing the motion of the particles was as follows:

(2.1) $$m\frac{\text{d}u_{\text{p}}}{\text{d}t}=F_{\text{D}}+mg,$$

where *m* is the mass of the particle and $u_{\text{p}}$ is the velocity of a particle subjected to a drag force $F_{\text{D}}$ and a gravity field ***g***. Details on the mathematical description of the drag force can be found in Harrington *et al*. (2006).

Approximately 30 000 locations uniformly distributed over the inlet were used to inject particles at the start of inspiration; particle deposition being independent of the number of locations used above 30 000 (increasing the number of particles tracked to 70 000 altered the deposition by less than 0.5%). The initial velocity of the particles was set to that of the local air velocity. If a particle encountered a wall of the structure, it was assumed to be deposited. If a particle reached the outlet of the model, it was assumed to have escaped the model. In both cases, the particle was no longer considered in the computation.

## 3. Results

### (a) Flow field

**Figure 2:**
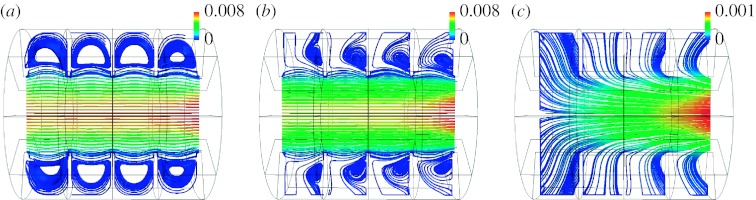
Flow pattern in a longitudinal section of the three-dimensional alveolated duct model with expanding walls 1 s after the beginning of a 2 s inspiration, i.e. at time *T*/4 where *T* is the breathing period. (*a*,*b*) Streamlines coloured by velocity magnitude (in 10^4^ cm s^−1^) in an alveolated duct representative of generation 18 of the Weibel symmetric lung model (Weibel 1963; Haefeli-Bleuer & Weibel 1988) in the stationary and MW cases, respectively, are shown. A parabolic velocity profile was imposed at the inlet of the duct and corresponded to a flow rate at the mouth of approximately 500 ml s^−1^. (*c*) Streamlines in the MW model representative of generation 23 of the Weibel symmetric model are shown. Flow was induced as a result of the motions of the alveolar walls. In all panels, flow was from right to left. Note the presence of significant radial flow in the expanding structures compared with the SW structure.

Figure 2 shows the flow pattern 1 s after the beginning of a 2 s inspiration. Figure 2*a*,*b* shows the flow field predicted for generation 18 in a section of the three-dimensional model for the stationary and MW cases, respectively, with flow being from right to left. In the SW structure, the flow field was characterized by curvilinear streamlines at alveolar openings. No bulk convective exchange existed between the central lumen and the surrounding alveoli; rather, a separation streamline was present at the mouth of the alveoli. A recirculating flow was positioned centrally in each alveolus with a velocity of approximately two orders of magnitude smaller than the mean lumen velocity. This flow regime was consistent with that seen in previous studies with non-moving boundaries and a low-Reynolds number flow (Tsuda *et al*. 1994*a*; Darquenne & Paiva 1996; Harrington *et al*. 2006).

Compared with the flow pattern in the SW structure (figure 2*a*), a significant portion of the flow in the central channel of the MW model entered the alveoli to satisfy mass balance (figure 2*b*). Alveolar recirculation was still present but was moved proximally compared with that in the SW model. Velocities in the alveoli were higher in the MW model than in the SW case. In the MW model, velocities in the alveoli were approximately one order of magnitude less than the mean lumen velocity compared with two orders of magnitude less in the SW model.

Flow predicted in the MW alveolar sac (generation 23) is shown in figure 2*c*. It largely differed from that in the proximal acinar duct (*Z*=18), and in the stationary cases in that the recirculation zones inside the alveolar cavities completely disappeared. Mean velocities in the alveolar cavities were only slightly less than mean lumen velocity with the alveolar flow being largely radial.

### (b) Particle deposition

**Figure 3:**
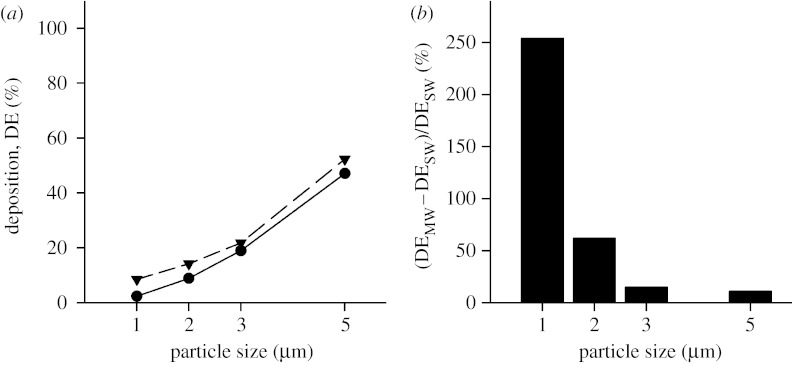
Deposition as a function of particle size in the three-dimensional alveolated duct representative of generation 18 of the Weibel model (*Z*=18). (*a*) Deposition for both the stationary wall (SW; circles) and the moving wall (MW; triangles) case is shown. (*b*) The percentage increase in deposition predicted in the MW structure compared with the SW structure is shown.

All simulations were performed with the duct being horizontal and with gravity acting downwards as shown in figure 1. The effect of gravity orientation on deposition has been studied previously (Tsuda *et al*. 1994*b*; Darquenne 2002; Harrington *et al*. 2006) and is not addressed here. Rather, this study focused on the effect of MW boundaries on predicting aerosol deposition. Figure 3*a* compares the predicted deposition of 1–5 μm diameter particles in the stationary and MW models of generation 18 of the Weibel model. Deposition varied from 2.4 to 47 per cent between 1 and 5 μm diameter particles in the SW case (figure 3*a*, closed circles) and from 8.5 to 52 per cent in the MW case (figure 3*a*, closed triangles). Deposition was always higher in the MW structure than in the SW case. While the differences appear small between the two models, when the percentage changes are considered, it is clear that, for small particles, a significant underestimation of deposition occurs when wall motion is neglected. Figure 3*b* displays the percentage increase in deposition between predictions obtained in the stationary and MW cases. The percentage increase was calculated as follows:

(3.1) $$\Delta \text{DE}(\%)=\frac{{\text{DE}}_{\text{MW}}-{\text{DE}}_{\text{SW}}}{{\text{DE}}_{\text{SW}}}\times 100,$$

where DE_MW_ and DE_SW_ are deposition predicted in the moving and SW cases, respectively. The percentage increase in deposition was the largest for small particles and varied from 254 per cent down to 11 per cent between 1 and 5 μm diameter particles.

**Figure 4:**
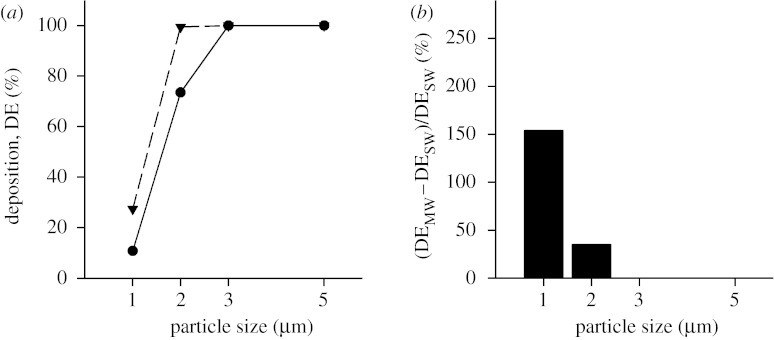
Deposition as a function of particle size in the three-dimensional alveolated duct representative of generation 23 of the Weibel model (*Z*=23). Data are shown in the same format as in figure 3. (*a*) Deposition for both the stationary wall (SW; circles) and the moving wall (MW; triangles) case. (*b*) The percentage increase in deposition predicted in the MW structure compared with the SW structure.

While a significant number of 5 μm diameter particles will reach the acinar region during tidal breathing, the number of particles that will reach the last generation is quite low; however, it is not zero. Data obtained for *Z*=23 are shown in figure 4 in the same format as in figure 3. Deposition reached 100 per cent for a particle diameter of 3 μm and larger in the SW case (figure 4*a*, closed circles) and for a particle diameter of 2 μm and larger in the MW case (figure 4*a*, closed triangles). For each particle size, the percentage increase in deposition (figure 4*b*) was less in simulations performed for *Z*=23 than that for *Z*=18, but still substantial for small particles.

## 4. Discussion

Until the mid-1990s, most of the model analyses describing aerosol transport and deposition in the lung were based on one-dimensional models of the lung. The early models estimated deposition in a model of the respiratory tract made up of a discrete number of morphometric regions (Landahl 1950; Beeckmans 1965; Yeh & Schum 1980; Hofmann *et al*. 1989). Deposition included that by Brownian diffusion, gravitational sedimentation and inertial impaction. Later models were based on a continuous description of aerosol transport in the lung (Taulbee & Yu 1975; Darquenne & Paiva 1994) where a one-dimensional convective–diffusive equation incorporating a term accounting for deposition was solved. The diffusive term incorporated both Brownian diffusion and an ‘apparent diffusion’ coefficient due to convective mixing. Convective mixing referred to all the mechanisms except Brownian motion that transferred particles from the inspired air into the resident air. However, the one-dimensional models as they existed did not provide a realistic description of the location of the actual sites of deposition (Darquenne & Paiva 1996). The main reason was that one-dimensional simulations rest on the implicit assumptions that the radial diffusion can be dealt with by the use of a single effective diffusion constant and that velocity and concentration are uniform over the cross section of the airways. Neither of these assumptions is valid in the complex structure of the lung, and especially not in the alveolar region. It therefore became apparent that multidimensional approaches were needed for more detailed and realistic information on aerosol behaviour in the acinar region of the lung.

### (a) Previous studies in models with rigid walls

Tsuda *et al*. (1994*a*,*b*) were the first to perform detailed computations of intra-acinar aerosol transport. Their model consisted of an infinitely long central channel surrounded by alveoli with circular shapes. Darquenne & Paiva (1996) performed similar two-dimensional simulations in a four-generation structure of the alveolar zone as well as three-dimensional simulations in a single alveolated duct. Both groups showed that the streamline pattern of the flow was very sensitive to alveolar duct geometry, in particular near the alveolar aperture. They showed that the presence of the alveolar septa contributed to the penetration of particles in the very periphery of the lung. Both groups also showed that particles did not deposit uniformly on the alveolar walls. Small particles (smaller than 0.5 μm) deposited mainly near the entrance of the alveoli, while the deposition of larger particles (0.5–5 μm) was mainly affected by the gravity field. Very large particle concentration inhomogeneities were therefore expected within any acinar duct and at any moment of the respiratory cycle. These two-dimensional models provided relevant and new information on aerosol transport in the alveolar cavities. However, these models ignored the bifurcation areas that connected the alveolated ducts between generations; this is a potentially important site of deposition as suggested by Brody & Roe (1983), who showed that a significant number of particles were deposited at alveolar duct bifurcations in the lungs of mice and rats that were exposed to aerosolized dust.

**Figure 5:**
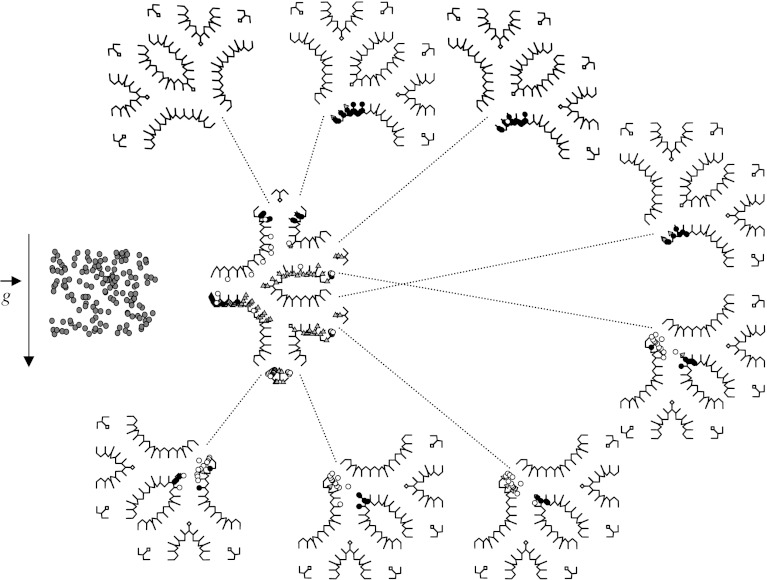
Symmetric six-generation structure of fully alveolated ducts of the adult human lung developed by Darquenne showing deposition pattern for 2 μm diameter particles after one breath cycle. The grey triangles and black circles represent the particles that were deposited during inspiration and expiration, respectively. The white and dark grey circles (shown outside the structure) represent the particles that did not deposit but remained in suspension or escaped the structure at the end of expiration, respectively. Adapted with permission from Darquenne (2001).

Subsequently, Darquenne (2001, 2002) developed a two-dimensional model of a symmetric six-generation structure of identical alveolated ducts that were formed by dichotomous branching in which a comprehensive study of aerosol deposition patterns for particle sizes that were most affected by gravitational sedimentation (0.5–5 μm) was performed (figure 5). Simulations were carried out for a typical breath pattern and up to five breath cycles were simulated. Simulations were performed for different orientations of the structure with respect to the gravity vector. These studies clearly showed that, for each particle size and structure orientation, there was a larger heterogeneity in deposition among ducts of the structure. In particular, considerable heterogeneity between ducts of the same generation was found in the distal generations. Local concentrations of deposited particles could be at least one order of magnitude larger than mean alveolar deposition. Another important observation was that a large number of the small particles (0.5, 1 and 2 μm) failed to exit the structure at the end of expiration (figure 5), even though they remained in suspension in the distal part of the structure. During subsequent breaths, these particles penetrated deeper into the lung, where they eventually deposited. The results from these simulations showed that inhaled particles reached regions of the lung which were beyond the inspired volume of air. These are important observations when one has to determine the potential effects of airborne pollutants on human health or the effectiveness of drugs administered by inhalation therapy.

Harrington *et al*. (2006) computed aerosol transport of 1–5 μm diameter particles in the first three-dimensional model of a fully alveolated bifurcation representative of two generations of the alveolar region of the lung. Their model improved over previous models with rigid walls as it explicitly represented the true surface area of alveoli, and the bifurcation areas between successive generations of fully alveolated ducts. They showed that the orientation of the structure had a significant effect on overall deposition, which varied by a factor of approximately 3 between the lowest and highest predicted depositions. More noteworthy is the fact that, comparing their results with predictions made in single isolated alveolated ducts, they showed the importance of modelling the bifurcation area because of the complex relationship between aerodynamic drag and gravitational sedimentation occurring in the bifurcation zone.

Because the Reynolds number in the acinar region of the lung is much less than unity, it has long been assumed that the acinar flow could be considered as a simple Poiseuille flow (i.e. a smooth parabolic flow profile along the central lumen). All the studies described so far (Tsuda *et al*. 1994*a*,*b*; Darquenne & Paiva 1996; Darquenne 2001, 2002; Harrington *et al*. 2006) show that this assumption is incorrect. Indeed, the presence of alveolar walls produces a flow pattern that is more complicated than a simple Poiseuille flow and that is characterized by curvilinear streamlines at the alveolar openings, and slow recirculating flow regions within the alveolar cavities. These complex flow patterns combined with typically small intrinsic properties of particles (inertia, sedimentation and diffusion) significantly affect aerosol transport and deposition in the lung periphery. One other aspect of acinar breathing that has not been addressed in any of these studies is the rhythmical expansion and contraction of the alveolar spaces that accompany each breath.

### (b) Previous models with moving walls

**Figure 6:**
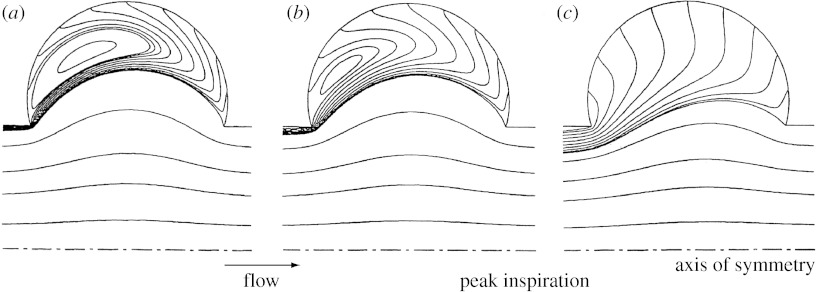
(*a*–*c*) Flow pattern in a model of a single alveolus with expanding walls developed by Tsuda *et al*. for three different ratios between alveolar and ductal flow with the ratio increasing from (*a*) the entrance of the acinus to (*c*) the periphery of the lung. Note that the smaller the ratio, the larger the recirculation zone. Adapted with permission from Tsuda *et al*. (1995).

Tsuda *et al*. (1995) extended their previous work in models with rigid walls to a model of a single alveolus with expanding walls, in which they computed the flow field. The geometric model consisted of a central channel surrounded by a torus to form an axisymmetric model of an alveolus (figure 6). They showed that the velocity field in the duct with MWs differed greatly from that in rigid-walled ducts. The fundamental difference was that the dividing separation streamline present at the entrance of the alveolar cavities in models with rigid walls completely disappeared in a model with MWs. Indeed, to satisfy mass balance, flow must necessarily enter and exit the alveolar cavity as it expands and contracts. A consequence of these observations is that, in an expanding structure, particles can penetrate the alveolar cavities not only as a result of intrinsic motions (diffusion and sedimentation) but also by convective transport, something not possible in a static structure.

An axisymmetric model made of nine tori was subsequently developed by the same group (Henry *et al*. 2002). This model addressed the cumulative effects of multiple alveoli on the flow pattern and therefore on the fate of inhaled aerosols. Massless particles were tracked in the model for different ratios of lumen flow versus alveolar flow (flow generated by the expansion and contraction of the cavities). Henry *et al*. (2002) showed that these particles followed irreversible trajectories and speculated that such kinematic irreversibility might be the dominant mechanism of aerosol transport in the periphery of the lung.

In a subsequent study, Haber *et al*. (2003) computed gravitational deposition of 0.5–2.5 μm diameter particles in a model of a single alveolus consisting of a rhythmically, self-similarly expanding, hemispherical cavity attached to a rhythmically stretching plane. They showed that, under equivalent shear flow conditions, deposition in an expanding and contracting alveolus was higher than that in a similar model with rigid walls, clearly demonstrating the role of alveolar wall motion in enhancing particle deposition in the alveolar cavity.

### (c) Effect of moving boundaries on aerosol transport in a three-dimensional fully alveolated duct

Building on the work of Haber *et al*. (2003) in a single alveolus, a three-dimensional model of a fully alveolated duct was developed in this study to further assess the effect of moving boundaries on predictions of particle deposition in the acinar region of the human lung. Our data show that the flow field was significantly different in the stationary and MW models in that the separation streamline present between the alveolar cavities and the lumen in the SW model (figure 2*a*) disappeared in the MW model (figure 2*b*,*c*). The very slow recirculation zone centred in the alveolar cavities of the SW model (figure 2*a*) either moved mouthward and had higher velocities (figure 2*b*) or completely vanished (figure 2*c*) in the MW model. The pattern of the alveolar flow field in the MW model was dependent on the ratio between the flow entering the alveolar cavities as a result of the expansion of the model (*Q*_A_) and that entering the lumen (*Q*_L_). In the more proximal generations of the acinus (*Z*=18), the ratio *Q*_A_/*Q*_L_ was relatively small (*Q*_A_/*Q*_L_=0.004) and, while it moved mouthward, a recirculation area still existed in the alveolar cavities. In the very periphery of the acinus (*Z*=23) where luminal flow is correspondingly lower, large values of *Q*_A_/*Q*_L_ (*Q*_A_/*Q*_L_=1) exist and the flow inside the alveolar cavities exhibits a radial pattern. These observations are in agreement with that of Tsuda *et al*. (1995) made in a single alveolus and that of Henry *et al*. (2002) made in an axisymmetric alveolated duct.

Several observations can be made from the deposition predictions made in both the stationary and MW models. First, in both types of models, deposition increased with increasing particle size. Second, for each particle size, deposition in proximal (*Z*=18) was less than that in peripheral acinar airways (*Z*=23) for both the stationary and MW models. Third, for a given particle size, deposition was higher in the MW model than that in the SW model both for *Z*=18 and for *Z*=23 but especially in the proximal acinar region (figure 3).

#### (i) Effect of particle size and residence time

Gravitational sedimentation is the main deposition mechanism of the particle size range considered in this study (1–5 μm; Darquenne 2006). According to Stokes' law, particles settle with a velocity (*v*_s_)

(4.1) $$v_{\text{s}}=\frac{\rho_{\text{p}}d_{\text{p}}^{2}}{18\mu }g,$$

where *ρ*_p_ is the particle density; *d*_p_ is the particle diameter; *μ* is the gas viscosity; and *g* is the gravitational acceleration. With the assumption of an unlimited source of aerosols, deposition by sedimentation should increase in a quadratic way with particle diameter *d*_p_. Our data show a quadratic increase only for predictions for *Z*=18. In the more peripheral model (*Z*=23), while deposition still increased with increasing particle size, the assumption of an unlimited source of aerosol was no longer valid because of the high deposition rate in the periphery of the acinus.

Deposition was higher in the very periphery of the lung (figure 4) than that in more proximal acinar airways (figure 3). These observations are similar to those made in previous studies by Tsuda *et al*. (1994*b*) and Harrington *et al*. (2006), and can be explained by the longer residence time of the particles in the distal compared with proximal acinar airways. Gravitational sedimentation, the main deposition mechanism of 1–5 μm diameter particles, is a time-dependent mechanism. Therefore, the longer a particle resides in an airspace, the more likely it will be to deposit.

#### (ii) Effect of alveolar expansion

The different nature of the flow patterns within the alveolar cavities between the stationary and MW models has a significant impact on the number of particles that deposit in the alveolated duct, with deposition being higher in the MW model than in the SW model in both proximal (*Z*=18) and distal (*Z*=23) acinar airways. The effect was larger for the smallest particles (1 μm) and for the proximal acinar airways. Deposition of 1 μm diameter particles more than tripled in the proximal acinar airways and more than doubled in the peripheral acinar airways between the stationary and MW models.

The only other study that looked at the effect of MWs on aerosol deposition is that by Haber *et al*. (2003) in a model of a single alveolus. They compared the deposition of 0.5 μm diameter particles between a cyclically expanding alveolus and one with rigid walls. Their simulations were made for a ratio *Q*_A_/*Q*_L_ of approximately 0.04, a value intermediate between our proximal and peripheral models. They found that deposition more than doubled when alveolar motion was included, a prediction in agreement with our data.

Haber *et al*. (2003) also determined the percentage of particles entering their model (alveolar-entering efficiency *η*) for different values of the ratio between the amplitudes of shear and expansion flows (*γ*), with large values of *γ* (aysmptotically equal to 3000) corresponding to alveolar flow near the entrance of the acinus and small values (less than 100) corresponding to alveolar flow in the acinar periphery. They showed that *η* increased as *γ* decreased and that, for *γ*<100, all particles that entered the alveolus deposited, suggesting higher deposition rates in the lung periphery than near the acinar entrance, in agreement with our data.

The increase in deposition between the stationary and MW models decreased as particle size increased (figures 3 and 4). In the MW model, particles can penetrate the alveolar cavities not only by gravitational sedimentation but also by convective transport. The average flow velocity at the alveoli openings in our model was approximately 475 μm s^−1^. Such velocity is more than one order of magnitude larger than the settling velocity of 1 μm diameter particles (approx. 33 μm s^−1^), but only approximately half the settling velocity of 5 μm diameter particles. Therefore, inclusion of moving boundaries will mostly affect small particles.

## 5. Conclusions

Computational fluid dynamics has been increasingly used over recent years to study aerosol transport and deposition in the lung. However, few of these studies have focused on the alveolar region of the lung. The first studies used two-dimensional and axisymmetric models with rigid walls ranging from a single alveolus to fully alveolated ducts. More recent studies have included models with moving boundaries. Even though Reynolds numbers are very low in the alveolar region of the lung (*Re*<1), these studies have shown that alveolar flow can be extremely complex due to the unique time-dependent geometry of the acinus. Aerosol transport and deposition are influenced by geometric characteristics, in particular at the level of the alveolar aperture, and there are large in homogeneities in deposition patterns within the acinar structure. The inclusion of alveolar septa increases the alveolar surface available for particles to deposit and therefore alters the concentration of deposited particles per unit area. The inclusion of MW boundaries increases convective exchange between the lumen and the surrounding alveolar cavities, which causes more particles to deposit. Such an increase in deposition due to MW boundaries is the greatest for small particles for which intrinsic motions are one order of magnitude less than mean flow velocities at the entrance of the alveoli. These observations strongly show that models that fail to incorporate alveolar wall motions may lead to incorrect predictions of aerosol deposition in the acinar region of the lung.

The results of simulations such as these go a long way towards elucidating a long-standing conundrum, namely the fact that model predictions of deposition, especially for small particles, are lower than those observed. This is the case in normal gravity (Tsuda *et al*. 2002) and in low gravity (Hoffman & Billingham 1975; Darquenne *et al*. 1997). While more realistic models ranging from one- to two- to three-dimensions somewhat improved such predictions, it is clear that, in the acinar region of the lung, MWs play a large and potentially dominant effect.

Acinar deposition is important in understanding the negative health consequences of particulate matter exposure (especially the small particles) as well as the effectiveness of targeted aerosolized delivery. At present, the three-dimensional MW models developed in this study are close to the limits of CFD capability, but future advances in these techniques will undoubtedly lead to further improvements in this area.
